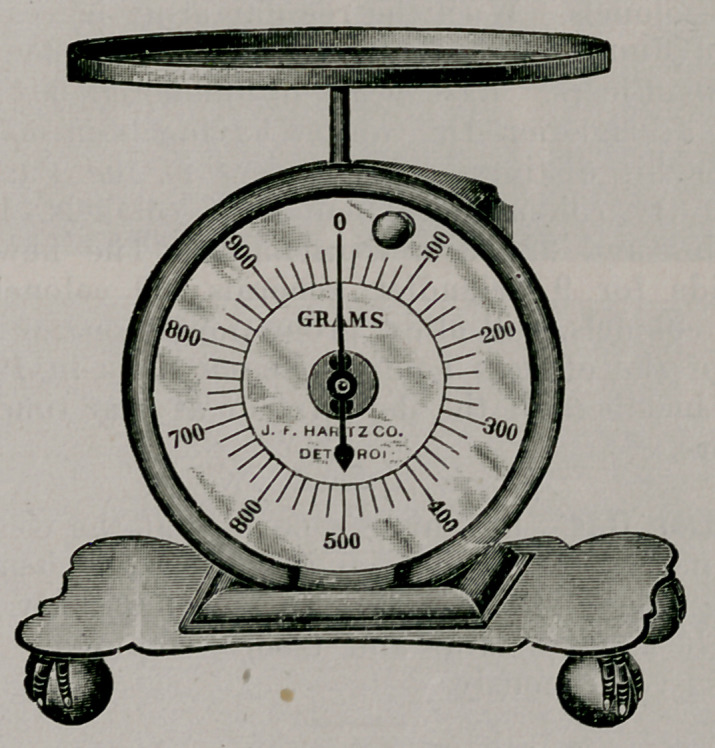# Book Reviews

**Published:** 1917-08

**Authors:** 


### BOOK REVIEWS

**Books mentioned may be inspected at and ordered through this office. So far as possible, books received in any month will be reviewed in the issue of the second month following. Pamphlets, quarterly and similar periodicals, reports, transactions, etc., will, as a rule, merely be mentioned.**

**Acute Poliomyelitis.** George Draper, M. D., N. Y., P. Blakiston's Son & Co., 149 pages, 19 illustrations, $1.50.

There is a ''foreword'' by Simon Flexner but this is followed by ''contents'' instead of inhold. Recent statistics are presented for 5496 N. Y. Cases. These included 94 cases in the age group 10-15; 32, 15-20; 40, 20-30; 25, 30-40; 7, 40-50, verifying the evidence of more general and less accurate statistics that this disease is really no more infantile than the average acute semelincident fever. Passive immunity has never been secured and only partial success has been achieved with experiments in active immunization of monkeys, several attempts with small or attenuated doses or virus having produced the disease itself. The Flexner-Noguchi organism is considered to be practically established as specific. The work is largely clinical but well summarized. The studies of types and of probable routes of infection—in either the anatomic or the geographic sense—are especially interesting.

**Pronostic De La Prostatectomie.** Dr. Victor Pauchet, Amiens. Illustrated pamphlet, based on 400 cases, with special reference to use of local anaesthesia.

The author advises operation in practically all cases except those obviously excluded by age and feebleness or those presenting mild symptoms easily controlled by catheterization. He has operated in about 80% of all cases encountered, the operations being about equally divided between those of one and of two sessions.

---

**Le Cabanon** (The Dungeon), Lucien Graux, published by A. Maloine & Fils, Paris. Paper cover, 196 pages, 4 francs.

This is a historic and clinical review of the use of incarceration and retentive devices in insane patients. The author favors their disuse and reports experience in various hospitals for the insane, showing the extent to which cells of different types are actually used.

**Diseases of the Stomach, Intestines and Pancreas.** By Robert Coleman Kemp, M. D., Professor of Gastro-intestinal Diseases at the Fordham University Medical School. Third edition, revised and enlarged. Octavo of 1096 pages, with 438 illustrations. Philadelphia and London: W. B. Saunders Company, 1917. Cloth, $7.00 net; Half Morocco $8.50 net.

The first edition of this work was a surprise to its readers. In many ways, it departed from the customary standards of discussion of gastro-enterologic conditions, introduced many novel methods, evinced originality, not as usual, in regard to one or a few developments of personal interests and research, but in regard to the general point of view and the whole conception. Yet careful reading showed that the author was neither an iconoclast nor a reconstructionist. Perhaps because the broadening influence of this author has been felt by more recent contributors to the general subject, the contrast does not seem so marked for the third edition. The work has been perfected in many details and has included various recent advances.

**Some Personal Recollections of Dr. (Edward Gamaliel) Janeway.** James Bayard Clark, G. P. Putnam's Sons. 36 pages, frontipiece portrait of Dr. Janeway, $1.

---

**Progressive Medicine,** edited by H. A. Hare and L. F. Appleman of Philadelphia; published by Lea & Febiger, Philadelphia and N. Y.; quarterly, $6 per year. Vol. 20, No. 2, June, 1917. 362 pages illustrated, reviewing recent progress in regard to Hernia (Wm. B. Coley); Surgery of the Abdomen (John C. A. Gerster); Gynaecology (John G. Clark); Diseases of the Blood, Diabetic and Metabolic Diseases, Thyroid, Spleen, Nutrition, Lymphatic System (Alfred Stengel); Ophthalmology (Edward Jackson).

This work is not only an index of periodic literature but a digest and critique of medicine in the light of the most recent and most authoritative studies. We call attention to a minor point that ought to be corrected: the confusion of volume and number on the title page.

---

**The Roentgen Diagnosis of Diseases of the Alimentary Canal.** By Russell D. Carman, M. D., Head of Section of Roentgenology, Division of Medicine, Mayo Clinic and Albert Miller, M. D., First Assistant in Roentgenology at the Mayo Clinic. Octavo of 558 pages with 504 original illustrations. Philadelphia and London: W. B. Saunders Company, 1917. Cloth $6.00 net; Half Morocco $7.50 net.

This is a most excellent, practical review of the subject, obviously based on large and discriminating experience. One general criticism might be made, purely from the didactic standpoint and in the interests of readers not themselves especially familiar with X-ray work. We would also qualify this criticism with the statement that it does not apply especially to the present book, indeed, less to it than to some others: Normal appearances, including what may be considered as normal variants, should, in each region or organ, be presented first and, perhaps be accompanied with diagrams or actual pictures. After having impressed on the mind of the student, the normal standards, each major disease or abnormality should be discussed systematically. It also assists the student to have all conditions shown from the front, and with right and left as in viewing the body, unless specifically stated to the contrary and otherwise represented for special reasons. Inexperienced students are also confused by differences according to the reproduction of positives and negatives, i.e. according to whether a relatively opaque area is shown as dark or light. Excepting purely diagrammatic delineations of areas marked on the exterior of the body, the authors seem to have used the latter method consistently. However, such a statement as accompanies Fig. 295: ''The (gall) stones are seen as dark areas lying in the more opaque bile,'' though occasioning no difficulty to one accustomed to X-ray studies, might easily be confusing to one unfamiliar with the subject, all the more so, if the reader did not realize that he was confused.

---

**Mr. Britling Sees it Through.** H. G. Wells, MacMillan Co., N. Y.

This is an unusual literary production. It is hardly a story, it has no central plot and not even the love episode is brought to a climax; the good and bad traits of the characters appear as in real life, without developing them into either heroes or villains. In a sketchy way, it reviews the history of the war and—how accurately we cannot judge—describes certain phases of English life. Mainly, it is a study of psychology—probably all the more valuable because not technical—as exemplified by the mental reflex to the changed conditions of war, by many persons of quite different type.

---

**Raymond. Sir Oliver Lodge.**

So much has been written about this collection of spiritualistic messages, that the book is somewhat disappointing even to one not a believer in such communications. The chief argument as to authenticity is the description of a group photograph of officers, including the deceased by a medium, before its existence was known to the family. It is not unusual to have such photographs made. Many of us not only fail to notify our family and friends of their existence but forget them ourselves. That a photograph including a dozen or so officers, including the son of a prominent parent known to be interested in spiritualism, should become known to mediums in advance of knowledge of its existence by the family, is not especially surprising. Indeed, if anything, the Mediums' Cooperative Assn. Ltd. or whatever it is called ought to have done a better detective job which would have avoided the slight inaccuracies in the description of the photograph admitted by the author. Possibly we are too greatly influenced by local pride but we would back mediums and controls within an afternoon's ride of Buffalo against those transmitting messages to Sir Oliver Lodge, for literary style, definiteness and thrill. The best part of the work is the letters of Raymond Lodge while in the flesh and doing active military duty. Like most other persons who have sent messages from the spirit world, he seems to have retrograded while the spiritual environment seems to be distinctly less interesting, useful and elevating than that with which we are acquainted.

**Roentgen Technic (Diagnostic).** Norman C. Prince, M. D., Omaha; C. V. Mosby Co., St. Louis, 140 pages, illustrated, $2.

This is a brief, practical treatise covering both the actual use of X-rays in diagnostic procedures and the development of plates, making of lantern slides, care of apparatus, etc. Chapter 5 on Positions and Exposures, is the longest and takes up in detail the various regional examinations, both for bone and visceral work. Chapter 6, on sinus injection, is the shortest—1 page—but by no means the least valuable. Chapter 8, Dark Room Procedures, throws light on a phase of X-ray work of which those not specializing in Roentgenology have almost no conception and which the man beginning to specialize in Roentgenology should study thoroughly.

**Diseases of the Genito-Urinary Organs and the Kidneys.** By Robert H. Greene, M. D., Professor of Genito-Urinary Surgery at the Fordham University, New York; and Harlow Brooks, M. D., Professor of Clinical Medicine, University and Bellevue Hospital Medical College. Fourth Edition. Thoroughly Revised. Octavo of 666 pages, 301 illustrations. Philadelphia and London: W. B. Saunders Company, 1917. Cloth. $5.50 net; Half Morocco, $7.00 net.

We have reviewed the earlier editions of this work which is so well known to the profession that we need say simply that it is accepted as a standard text book of comprehensive scope.

**28th Annual Archaeologic Report,** Appendix to the Report of the Minister of Education, Ontario, by Dr. Rowland B. Orr, Director of Provincial Museum, Toronto, 1916. 114 pages, illustrated.

The article on the Ape Man by Rev. W. R. Harris, D. D., LL.D., is of special interest to our readers, being a critical review of certain modern claims of discovery of primitive bones and of the reconstructions of early man, made from them.

**Traumatic Surgery** by John J. Moorehead, M. D., F. A. C. S. Adjunct Professor of Surgery in the New York Post-Graduate School and Hospital. Octavo volume of 760 pages with

522 original illustrations. Philadelphia and London: W. B. Saunders Company, 1917. Cloth, $6.50 net. Half Morocco, $8.00 net.

This work, while not essentially one on military surgery, incorporates the lessons of the present war and, so far as can be judged, adequately provides for preparation for duties in the field. It includes sections on traumatism from electricity, X-rays, etc., and on medico-legal problems, always a source of special anxiety to the surgeon. While systematic in the usual sense, its arrangement and indexing facilitate reference in emergency.

---

**Index of Differential Diagnosis of Main Symptoms,** by various writers. Edited by Herbert French, M. A., F. R. C. P., London. Published by Wm. Wood & Co., N. Y. 912 pages, 37 colored plates and over 300 illustrations in text. $10.

The major part of the work is arranged alphabetically by names of conditions, such as ascites, pain, wheals, pigmentation or of organs under which general conditions as of enlargement and gross alteration are easily noted. Disease names are included only when they also refer to obvious symptoms, as anaemia, hemiplegia, etc. Indeed, it is difficult to discriminate sharply between names of diseases and of clinical conditions of a clinical nature. This is supplemented with an index, still more comprehensive but referring to parts of the index by heavy type. The text is by no means confined to differential tables—which are almost always superficial and somewhat inaccurate—but includes a very good treatise on clinical conditions, entering into pathogeny and even etiology whenever necessary for clearness of conception. The work is available not only for immediate reference but, perhaps even more for training in habits of accurate diagnosis. Instead of leading to a snap diagnosis or inculcating superficial methods of naming diseases, this work rather broadens the conception of the reader and induces him to reflection as to the essential nature of processes brought to his attention by symptoms.

---

**Facts and Fallacies of Compulsory Health Insurance.** Frederick L. Hoffman, LL.D., Statistician Prudential Life Ins. Co., 101 pages.

This does not lend itself well to a brief abstract but deserves reading throughout. The claims commonly made for the success of compulsory health insurance in Germany are disputed, on the basis of comparison of American and German longevity, death, and suicide rates.

---

**Diseases of the Stomach.** Max Einhorn, M. D., New York, Wm. Wood & Co., 6th edition, 59 pages, 128 illustrations.

This edition has been considerably enlarged and modified to correspond to the developments of gastro-enterology. While conforming to the standards established for a comprehensive treatise, it deals to a considerable degree with the many ingenious devices originated by the author, among which may be mentioned the convenient assemblage of tubes for estimating pepsin, the stomach spray, insufflator and bucket, the duodenal tube and modification of the thread method, endoscopes, applicators, etc., including that for the use of radium and many others. The work, while limited by title to the stomach, necessarily includes the consideration of many associated disorders, though their discussion is not carried beyond the point requisite. Considerable attention is given to X-rays but we suggest that the first sentence under this heading should be changed. The mention of four men as having "succeeded in taking good Roentgenographs" is, under present conditions, a very mild statement and the implication is historically incorrect, so far as priority is concerned. It is difficult to single out sections for special commendation but the critical ability of the author is particularly manifested in those on ulcer, cancer and ischochymia and a careful reading of these sections would not only tend to correct several quite general misconceptions but to prevent unnecessary surgery and to expedite operation when it is necessary. The author has also chosen the happy mean between the early exaggeration of the importance of intubation and chemic tests and the present tendency in some quarters to ignore these matters simply because of their familiarity. Beyond any single item that might be selected for praise, is to be emphasized the author's rare combination of practicality with thorough scientific acumen.

---

**Eye, Ear, Nose and Throat.** A Manual for Students and Practitioners, Howard Charles Ballenger, M. D. and A. G. Wippern, M. D., Chicago. Lea & Febiger, Philadelphia. 2d edition, 524 pages, 8 colored plates, 180 illustrations, $3.50.

This is bound to correspond in pattern but not in color, to a series issued by this firm several years, covering various branches in medicine, though each book is complete in itself.

The present work has been thoroughly revised and is most excellent in its arrangement and text, being of scope intermediate between brief reviews and cyclopaedic works. Fig 8, a reproduction of a test card, needs a new plate.

---

### Review of Instrument.

**Dietetic Balance. J. F. Hartz Co.,** Detroit. This is a convenient spring scale with pan, indicating weights in units of 10 grams, up to 500. The dial is set by rotating so that paper, dishes, etc., required to hold non-solid or greasy foods, can be allowed for, and the indicator can be set at zero beyond the weight of container. One of the greatest practical obstacles to dietetic therapeutics and even economic studies is that very few persons, even physicians, have any conception of actual quantities in regard to foods. Knowing the weight of an article of food tables are available to calculate quite accurately, the content either in proteid, carbohydrate and fat, or the calories. Without this knowledge, dietetics is a combination of guess work and aerothermia, so far as quantitation is concerned. Even knowledge of waste, as skin and cores of fruit, trimmings of meat, etc., is often important. This scale also familiarizes the user with the metric system. It can be used for determining postage and for various other practical purposes where heavy weights are not involved.

## Figures and Tables

**Figure f1:**